# Supplementary material for: Assessing the Impact of Influenza Vaccination Timing on Experimental Arthritis: Effects on Disease Progression and Inflammatory Biomarkers
Source: Int J Mol Sci. 2024 Mar 14;25(6):3292. doi: 10.3390/ijms25063292 (PMC10969894; doi:10.3390/ijms25063292)
Supplement: Supplementary file 1 [file ijms-25-03292-s001.zip › ijms-2857108-supplementary.pdf]

Table S1. a, Values of Figure 1 expressed as mean ± S.E.M **b,** p-values of Figure 1

a,

	Baseline control	CFA control	CFA early	CFA late	Flu early	Flu late
N	6	7	7	7	7	6
Unit	Mean±S.E.M					
%	11,12± 4,681	5,665±3,164	4,882±5,438	6,476± 3,309	10,79± 2,719	11,41± 4,794
gram	313,4± 13,19	295,9±8,866	296,1±15,28	299,6±18,57	311,2± 7,641	314,4± 9,287

b,

Day		day 2	day 9	day 21
Baseline Control vs. Flu early	p-values	ns	ns	ns
Baseline Control vs. Flu late		ns	ns	ns
Baseline Control vs. CFA control		ns	ns	ns
Baseline Control vs. CFA+Flu early		ns	0,0156	0,0429
Baseline Control vs. CFA+Flu late		ns	0,046	ns
Flu early vs. Flu late		ns	ns	ns
Flu early vs. CFA control		ns	ns	ns
Flu early vs. CFA+Flu early		ns	ns	0,0048
Flu early vs. CFA+Flu late		ns	ns	ns
Flu late vs. CFA control		ns	ns	ns
Flu late vs. CFA+Flu early		0,0474	0,0079	0,005
Flu late vs. CFA+Flu late		ns	0,0104	ns
CFA control vs. CFA+Flu early		ns	ns	ns
CFA control vs. CFA+Flu late		ns	ns	ns
CFA+Flu early vs. CFA+Flu late		ns	ns	ns

Table S2. a, Values expressed as mean ± S.E.M for Figures 2 A and B **b,** p-values for Figure 2 A **c,** for Figure 2 B

a,

		Baseline Control	CFA control	CFA early	CFA late	Flu early	Flu late
N		6	6	6	6	6	6
examination	Unit						
plethysmometry	%	6,711± 3,301	56,53± 11,89	60,41± 12,61	67,23± 13,87	6,371± 4,192	8,53± 4,460
dynamic plantar aesthesiometer	%	- 1,384± 0,9723	-46,99±23,95	-38,33±20,67	-40,88±20,43	-1,662±0,8229	-0,6276±0,6858

b,

Day	0	1	4	11	18
Baseline Control vs. Flu early	ns	ns	ns	ns	ns
Baseline Control vs. Flu late	ns	ns	ns	ns	ns
Baseline Control vs. CFA control	0,0168	0,0378	0,0034	0,0202	ns
Baseline Control vs. CFA+ Flu early	0,0169	0,02	<0,0001	0,0065	ns
Baseline Control vs. CFA+Flu late	0,0031	0,0004	0,0001	0,0082	0,0389
Flu early vs. Flu late	ns	ns	ns	ns	ns
Flu early vs. CFA control	0,0182	0,0089	0,0039	0,025	ns
Flu early vs. CFA+ Flu early	0,0182	0,0046	<0,0001	0,0081	ns
Flu early vs. CFA+Flu late	0,0028	0,0002	0,0004	0,01	ns
Flu late vs. CFA control	0,0223	0,0157	0,0025	0,0325	ns
Flu late vs. CFA+ Flu early	0,0218	0,0081	<0,0001	0,0107	ns
Flu late vs. CFA+Flu late	0,0031	0,0002	0,0001	0,0125	ns
CFA control vs. CFA+ Flu early	ns	ns	ns	ns	ns
CFA control vs. CFA+Flu late	ns	ns	ns	ns	ns
CFA+ Flu early vs. CFA+Flu late	ns	ns	ns	ns	ns

c,

Day	1	4	7	14	21
Baseline Control vs. Flu early	ns	ns	ns	ns	ns
Baseline Control vs. Flu late	ns	ns	ns	ns	ns
Baseline Control vs. CFA control	<0,0001	0,0002	<0,0001	<0,0001	<0,0001
Baseline Control vs. CFA+ Flu early	0,001	0,0292	0,0002	0,0008	<0,0001
Baseline Control vs. CFA+Flu late	0,0003	<0,0001	0,0002	0,0104	0,0022
Flu early vs. Flu late	ns	ns	ns	ns	ns
Flu early vs. CFA control	0,0001	0,0003	<0,0001	<0,0001	<0,0001
Flu early vs. CFA+ Flu early	0,0012	ns	<0,0001	0,0007	<0,0001
Flu early vs. CFA+Flu late	0,0004	0,0003	0,0002	0,0094	0,0029
Flu late vs. CFA control	<0,0001	0,0002	<0,0001	<0,0001	<0,0001
Flu late vs. CFA+ Flu early	0,0011	0,0282	<0,0001	0,0004	<0,0001
Flu late vs. CFA+Flu late	0,0003	<0,0001	0,0002	0,006	0,0021
CFA control vs. CFA+ Flu early	ns	ns	ns	ns	ns
CFA control vs. CFA+Flu late	ns	ns	ns	ns	ns
CFA+ Flu early vs. CFA+Flu late	ns	ns	ns	ns	ns

Table S3: a, Raw data of Figures 3 and 4 **b**, results of Tukey's multiple comparisons test for Figures 3 and 4

a,

		total photon flux/s Mean ± S.E.M	photons/s/cm2/sr]/[µW/cm2 Mean ± S.E.M
Day 2	Baseline control	107050 ± 24065	32616666667 ± 1943436247
	CFA control	414429 ± 67731	76985714286 ± 4289268368

	CFA FLU early	508429 ± 64596	82233333333 ± 4831953826
	Flu Early	88529 ± 14916	28457142857 ± 871467603
	CFA FLU late	468429 ± 61927	90000000000 ± 6390804406
	FLU late	121650 ± 24377	32700000000 ± 2164563081
Day 9	Baseline control	68950 ± 7906	31166666667 ± 1115546702
	CFA Control	700943 ± 216903	114500000000 ± 12332369332
	CFA FLU early	631000 ± 109710	102442857143 ± 8037911869
	FLU early	83283 ± 10310	32783333333 ± 1210899574
	CFA FLU late	804571 ± 185513	106883333333 ± 14120916322
	FLU late	110850 ± 22138	33283333333 ± 1613157704

b,

	neutrophil MPO activity		plasma leakage	
		p-values		p-values
Control D2 vs. CFA D2	ns	0,6059	***	0,0004
Control D2 vs. CFA FLU early D2	ns	0,2134	****	<0,0001
Control D2 vs. Flu Early D2	ns	>0,9999	ns	>0,9999
Control D2 vs. CFA FLU late D2	ns	0,3575	****	<0,0001
Control D2 vs. FLU late D2	ns	>0,9999	ns	>0,9999
Control D2 vs. Control D9	ns	>0,9999	ns	>0,9999
Control D2 vs. CFA D9	**	0,0058	****	<0,0001
Control D2 vs. CFA FLU early D9	*	0,0256	****	<0,0001
Control D2 vs. FLU early D9	ns	>0,9999	ns	>0,9999
Control D2 vs. CFA FLU late D9	***	0,0005	****	<0,0001
Control D2 vs. FLU late D9	ns	>0,9999	ns	>0,9999
CFA D2 vs. CFA FLU early D2	ns	>0,9999	ns	>0,9999
CFA D2 vs. Flu Early D2	ns	0,455	****	<0,0001
CFA D2 vs. CFA FLU late D2	ns	>0,9999	ns	0,9356
CFA D2 vs. FLU late D2	ns	0,6745	***	0,0004
CFA D2 vs. Control D9	ns	0,4265	***	0,0002
CFA D2 vs. CFA D9	ns	0,6492	**	0,0052
CFA D2 vs. CFA FLU early D9	ns	0,9171	ns	0,1547
CFA D2 vs. FLU early D9	ns	0,4926	***	0,0004
CFA D2 vs. CFA FLU late D9	ns	0,1996	ns	0,0624
CFA D2 vs. FLU late D9	ns	0,624	***	0,0005
CFA FLU early D2 vs. Flu Early D2	ns	0,1243	****	<0,0001
CFA FLU early D2 vs. CFA FLU late D2	ns	>0,9999	ns	0,9993
CFA FLU early D2 vs. FLU late D2	ns	0,2607	****	<0,0001
CFA FLU early D2 vs. Control D9	ns	0,1195	****	<0,0001
CFA FLU early D2 vs. CFA D9	ns	0,9622	*	0,0439
CFA FLU early D2 vs. CFA FLU early D9	ns	0,9991	ns	0,5310

CFA FLU early D2 vs. FLU early D9	ns	0,1501	***	0,0001
CFA FLU early D2 vs. CFA FLU late D9	ns	0,6018	ns	0,2865
CFA FLU early D2 vs. FLU late D9	ns	0,2251	***	0,0001
Flu Early D2 vs. CFA FLU late D2	ns	0,232	****	<0,0001
Flu Early D2 vs. FLU late D2	ns	>0,9999	ns	>0,9999
Flu Early D2 vs. Control D9	ns	>0,9999	ns	>0,9999
Flu Early D2 vs. CFA D9	**	0,0021	****	<0,0001
Flu Early D2 vs. CFA FLU early D9	*	0,011	****	<0,0001
Flu Early D2 vs. FLU early D9	ns	>0,9999	ns	>0,9999
Flu Early D2 vs. CFA FLU late D9	***	0,0001	****	<0,0001
Flu Early D2 vs. FLU late D9	ns	>0,9999	ns	>0,9999
CFA FLU late D2 vs. FLU late D2	ns	0,4206	****	<0,0001
CFA FLU late D2 vs. Control D9	ns	0,2192	****	<0,0001
CFA FLU late D2 vs. CFA D9	ns	0,8731	ns	0,2450
CFA FLU late D2 vs. CFA FLU early D9	ns	0,9895	ns	0,9523
CFA FLU late D2 vs. FLU early D9	ns	0,2663	****	<0,0001
CFA FLU late D2 vs. CFA FLU late D9	ns	0,4069	ns	0,7738
CFA FLU late D2 vs. FLU late D9	ns	0,3735	****	<0,0001
FLU late D2 vs. Control D9	ns	>0,9999	ns	>0,9999
FLU late D2 vs. CFA D9	**	0,008	****	<0,0001
FLU late D2 vs. CFA FLU early D9	*	0,0341	****	<0,0001
FLU late D2 vs. FLU early D9	ns	>0,9999	ns	>0,9999
FLU late D2 vs. CFA FLU late D9	***	0,0007	****	<0,0001
FLU late D2 vs. FLU late D9	ns	>0,9999	ns	>0,9999
Control D9 vs. CFA D9	**	0,0024	****	<0,0001
Control D9 vs. CFA FLU early D9	*	0,0116	****	<0,0001
Control D9 vs. FLU early D9	ns	>0,9999	ns	>0,9999
Control D9 vs. CFA FLU late D9	***	0,0002	****	<0,0001
Control D9 vs. FLU late D9	ns	>0,9999	ns	>0,9999
CFA D9 vs. CFA FLU early D9	ns	>0,9999	ns	0,9712
CFA D9 vs. FLU early D9	**	0,0033	****	<0,0001
CFA D9 vs. CFA FLU late D9	ns	0,9998	ns	0,9996
CFA D9 vs. FLU late D9	**	0,0063	****	<0,0001
CFA FLU early D9 vs. FLU early D9	*	0,0157	****	<0,0001
CFA FLU early D9 vs. CFA FLU late D9	ns	0,9824	ns	>0,9999
CFA FLU early D9 vs. FLU late D9	*	0,0276	****	<0,0001
FLU early D9 vs. CFA FLU late D9	***	0,0003	****	<0,0001
FLU early D9 vs. FLU late D9	ns	>0,9999	ns	>0,9999
CFA FLU late D9 vs. FLU late D9	***	0,0005	****	<0,0001

Table S4: Raw expression data of western blot analysis (Figure 5) expressed as mean ± S.E.M

	N	Examined protein	MPO	MMP9	HMOX1	TNF-alpha
Baseline Control	5	Mean ± SD	0±0	0±0	97,28±43,21	100±0
CFA control	5		18,14± 3,344	85,56± 21,87	78,56±24,44	128,4± 63,54
CFA early	5		141,3± 63,15	91,93± 9,933	98,17±19,44	55,87± 15,35
CFA late	5		215,9± 33,05	133,2± 10,78	155,1±29,44	243,9± 107,2
Flu early	5		0±0	4,078± 4,078	41,12±32,81	89,10± 34,11
Flu late	5		0±0	0±0	6,311±3,318	54,12± 21,16
						N=3

Table S5: p-values of parameters in Figure 5

	N	Examined protein	MPO	MMP9	HMOX1	TNF-alpha N=3
Baseline control vs. CFA control	5	p-values	ns	ns	ns	ns
Baseline control vs. CFA early	5		0,0236	ns	ns	ns
Baseline control vs. CFA late	5		0,0003	0,0038	ns	ns
Baseline control vs. Flu early	5		ns	ns	ns	ns
Baseline control vs. Flu late	5		ns	ns	ns	ns
CFA control vs. CFA early	5		ns	ns	ns	ns
CFA control vs. CFA late	5		0,0009	ns	ns	ns
CFA control vs. Flu early	5		ns	ns	ns	ns
CFA control vs. Flu late	5		ns	ns	ns	ns
CFA early vs. CFA late	5		ns	ns	ns	ns
CFA early vs. Flu early	5		0,0236	ns	ns	ns
CFA early vs. Flu late	5		0,0236	ns	ns	ns
CFA late vs. Flu early	5		0,0003	0,0012	ns	ns
CFA late vs. Flu late	5		0,0003	0,0038	0,012	ns
Flu early vs. Flu late	5		ns	ns	ns	ns

Table S6. Data for Figure 1S and 2S

	nMPO activity		vascular leakage	
	total photon flux/s Mean	± S.E.M.	photons/s/cm2/sr]/[µW/cm2 Mean	± S.E.M.
Sham control D2 contralateral	95867	22855	31883333333	1797297354
Sham control D2 treated	107050	24065	32616666667	1943436247
CFA control D2 contralateral	74857	3955	**** 33928571429	1651509308
CFA control D2 treated	414429	67731	76985714286	4289268368
CFA Flu early D2 contralateral	84829	9748	**** 31550000000	876641318

CFA Flu early D2 treated	508429	64596	82233333333	4831953826
Sham Flu early D2 contralateral	82857	9523	28283333333	574697988
Sham Flu early D2 treated	88529	14916	28457142857	871467603
Sham control early D2 contralateral	98617	23244	32566666667	862811940
Sham control D9 treated	68950	7906	31166666667	1115546702
CFA control D9 contralateral	**** 117929	14503	**** 39300000000	2677312085
CFA control D9 treated	700943	216903	1,145E+11	12332369332
CFA Flu early D9 contralateral	106714	11823	**** 40242857143	2252919497
CFA Flu early D9 treated	83283	10310	1,02443E+11	8037911869
Sham Flu early D9contralateral	**** 90050	9803	32616666667	781629352
Sham Flu early D9 treated	631000	109710	32783333333	1210899574
CFA Flu late D2 contralateral	135429	11082	**** 38971428571	2521877068
CFA Flu late D2 treated	468429	61927	90000000000	6390804406
Sham Flu late D2 contralateral	95600	19186	34716666667	2596589644
Sham Flu late D2 treated	121650	24377	32700000000	2164563081
CFA Flu late D9 contralateral	**** 110843	13685	**** 32566666667	862811940
CFA Flu late D9 treated	804571	185513	1,06883E+11	14120916322
Sham Flu late D9 contralateral	78967	12270	32350000000	1380519226
Sham Flu late D9 treated	110850	22138	33283333333	1613157704
**** : contra vs. ipsi p<0,0001				

Figure S1. Comparison of the effect of Freund's complete adjuvant and **a**, early influenza vaccination or **b**, late influenza vaccination on myeloperoxidase activity in the treated and the contralateral paws of the animals.

a,

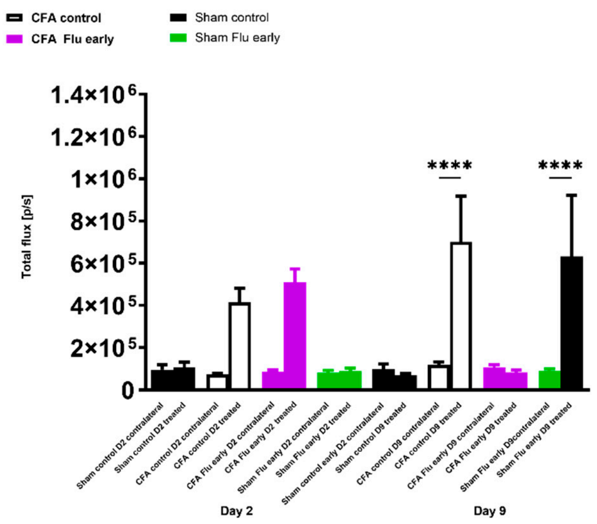

b,

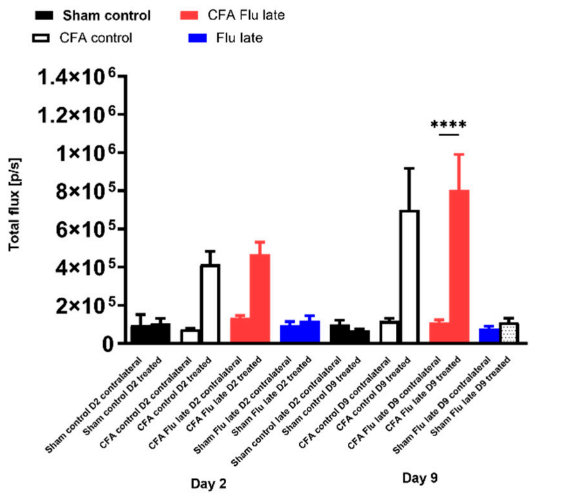

Figure S2. Comparison of the effect of Freund's complete adjuvant and **a**, early influenza vaccination or **b**, late influenza vaccination on vascular leakage in the treated and the contralateral paws of the animals.

a,

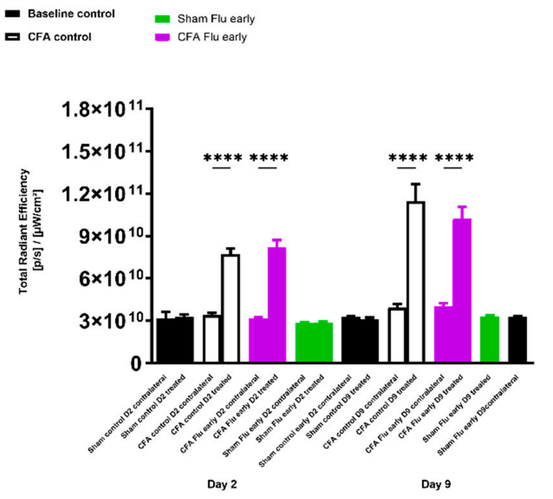

b,

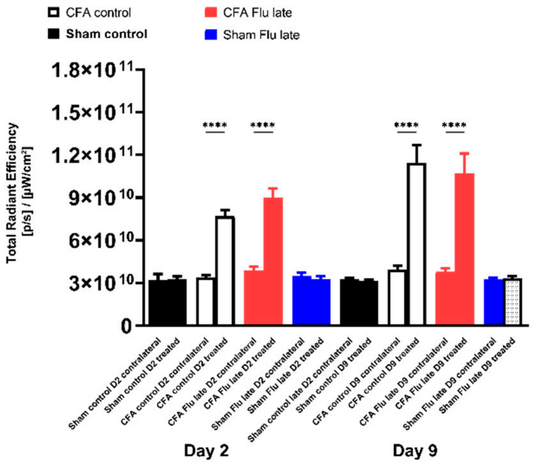

Figure S3. Microscopic Morphometry of Joint Inflammation

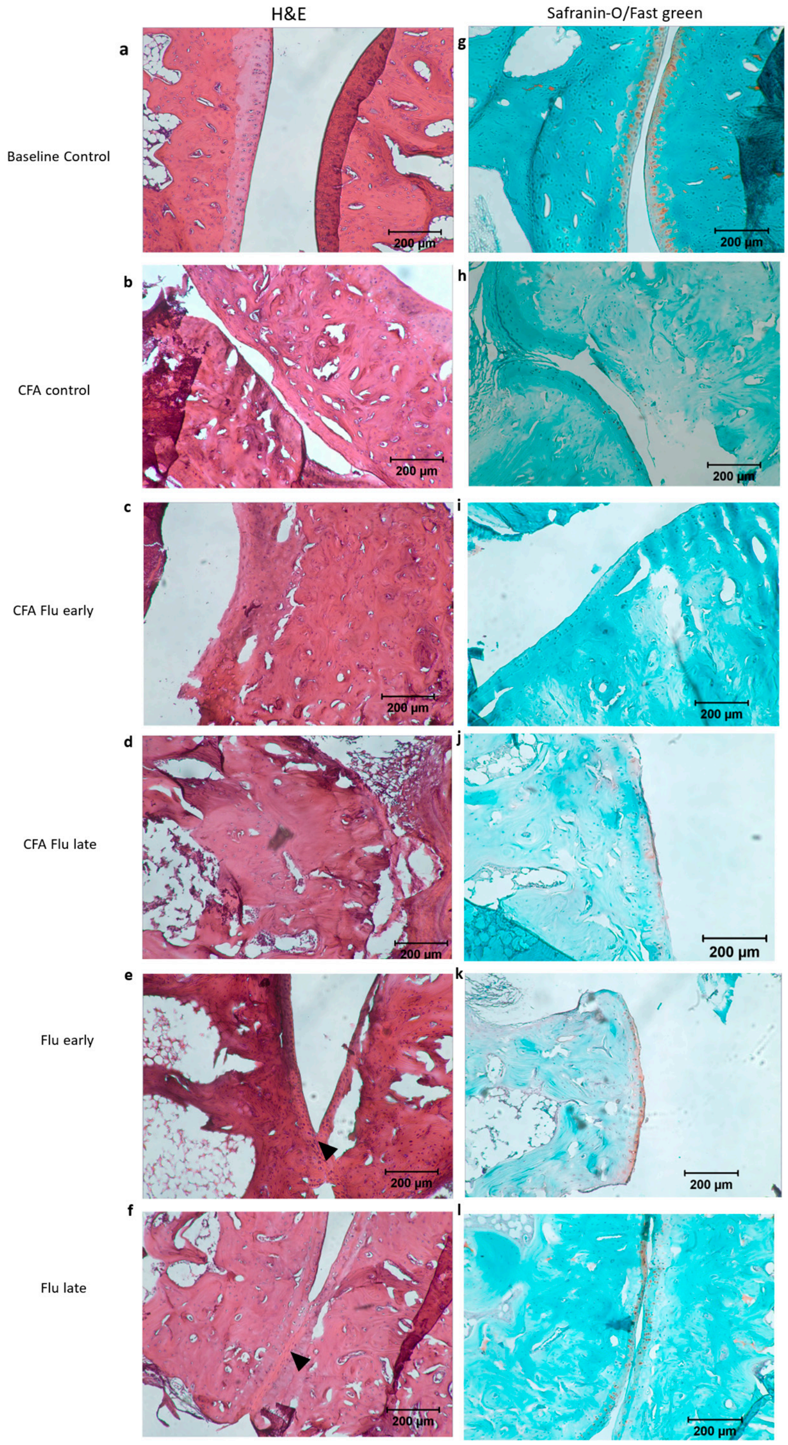

Histologic samples of the experimental animals. **a, g** Baseline control **b**, **h** CFA control **c**, **i** CFA Flu early **d,j** CFA Flu late **e**, **k** Flu early **f**, **l** Flu late from rats injected with the FluArt vaccine on the seventh day of the experiment. Samples a-f stained with H&E, g-l stained with Safranin-O/Fast Green. (magnification: 400x) Dark triangles mark the accreted cartilaginous tissue of the joint.

When analyzing the histological samples the signs of inflammation were detectable on the specimens taken from CFA-treated animals (Figure 7. d,e,f,j,k,l)). Mononuclear cell infiltration, bone destruction, and invading synovium were also observable in the groups where the animals were also administered influenza vaccine (Figure 7. e,k,f,l). By the samples of animals from the group Flu early and Flu late a special aberration was detected, the surface of the joint accreted (Figure 7. b,c)
